# Interaction between two rice mitogen activated protein kinases and its possible role in plant defense

**DOI:** 10.1186/1471-2229-13-121

**Published:** 2013-08-28

**Authors:** Arsheed H Sheikh, Badmi Raghuram, Siddhi K Jalmi, Dhammaprakash P Wankhede, Pallavi Singh, Alok K Sinha

**Affiliations:** 1 National Institute of Plant Genome Research, Aruna Asaf Ali Road, New Delhi 110067, India

**Keywords:** MAP kinase, Protein-protein interaction, *Pseudomonas syringae pv. tabaci*, Rice, Yeast two-hybrid assay

## Abstract

**Background:**

The canonical mitogen activated protein kinase (MAPK) signaling pathway plays a vital role in carrying out the normal growth and development of the plant. The pathway, connecting the upstreams signal with the downstream target is considered to be linear, mostly starting with a MAPKKK and ending in a MAPK.

**Results:**

Here we report a novel interaction between two rice MAPKs, OsMPK20-4 and OsMPK3 suggesting the complex nature of the pathway rather than a linear one at individual steps. The interaction between OsMPK20-4 and OsMPK3 found by yeast two-hybrid analysis was confirmed *in planta* by co-immunoprecipitation and fluorescence resonance energy transfer (FRET) assays. The interaction is specific and is phosphorylation independent. The results suggest a role of the interaction between OsMPK20-4 and OsMPK3 in basic plant defense.

**Conclusions:**

The current novel work showing the physical interaction between two plant MAPKs, OsMPK20-4 and OsMPK3 is the diversion from the dogma of a typical MAPK cascade thereby opening a new dimension to the MAPK signal transduction.

## Background

Mitogen activated protein kinase (MAPK) signaling cascade plays a vital role in conferring resistance to the sessile plants besides coordinating the normal growth and developmental cues. It is one of the primary and evolutionary conserved signaling cascades possibly derived from animal ERK-related lineage [1]. A canonical MAP kinase pathway minimally consists of a three tier linear phospho-transfer module namely MAPKKK-MAPKK-MAPK which connects diverse developmental and defense signals to the appropriate transcriptional response. MAPKs are phosphorylated at a conserved T-E-Y or T-D-Y motif by upstream MAPKKs which lead to their activation [2]. The downstream substrates of activated MAPKs include transcription factors, enzymes or other proteins both in the cytoplasm and the nucleus [3]. The sequences of *Arabidopsis* MAPK substrates contain serine or threonine followed by proline [S/TP] which is considered as the minimal consensus motif for phosphorylation by MAPK [4].

The main illustrations about plant MAPKs come from eudicot plant *Arabidopsis* which contains 20 MAPKs divided into four groups A-D [5]. Groups A, B and C have the ‘TEY’ motif, whereas group D contains ‘TDY’ motif between subdomains VII-VIII, a characteristic feature of MAPKs [6]. The most extensively studied group A MAPKs from *Arabidopsis,* AtMPK3 and AtMPK6 are central positive mediators of plant innate immunity [7,8] besides priming plants for the subsequent infections [9]. Group B MAPK, AtMPK4 acts as a negative regulator of plant systemic acquired resistance [10]. *Arabidopsis* MAPKs are also known to control basic physiological and developmental processes ranging from ethylene biosynthesis and signaling [11-13], stomatal development and response [14,15], cortical microtubule function [16], ovule development [17] to senescence [18].

The information about the MAPK gene family, their function and regulation in economically important cereal crop rice (*Oryza sativa*) is still scanty compared to its model eudicot plant *Arabidopsis*. Rice has 15 MAPKs (OsMPKs) ranging from molecular masses of 42–79 kDa with conserved protein kinase domains I–XI and an activation T-loop of either TEY or TDY motifs [5]. Interestingly rice has more TDY motif containing group D MAPKs (10 members) than with the TEY motif (5 members) which is in contrast with *Arabidopsis* MAPKs. OsMAPKs in TDY subgroup have three to four extra amino acid insertions near the activation loop as compared to OsMAPKs of the TEY subgroups. OsMAPKs belonging to the TEY subgroup have a C-terminal common docking (CD) domain that functions as a docking site for MAPKKs, while members of the TDY subgroup lack this CD domain but have a relatively long C-terminal region [19].

Out of 15 OsMAPKs in rice only a few have been characterized so far. For example OsMPK3 (previously named as OsBIMK1, OsMAP1, OsMSRMK2, OsMAPK2 or OsMPK5) and OsMPK7 (previously named as OsMAPK4, OsMSRMK3) were shown to be induced by various biotic and abiotic stresses [20-24]. Similarly OsMPK17-1 (OsBWMK1), OsMPK6 (OsMAPK6, OsSIPK) and OsMPK4 (OsMPK2) were found to be involved in plant defense response [25-27].

*OsMPK20-4* (Acc. No. DQ826425.1) (previously named as OsWJUMK, OsMPK8 and OsMPKG1) is one of the important rice MAPK members encoding a 569 amino acid long protein [28]. OsMPK20-4 is composed of an N-terminal kinase domain (KD) and an unusually long C-terminal extension region but lacks the common docking (CD) domain. Unlike most other plant MAPKs, the KD region of OsMPK20-4 carries a TDY phosphorylation motif instead of TEY, a sequence essential in MAPK activation. OsMPK20-4 has been classified as Group D MAPK and share the highest similarity with AtMPK20 [5,19]. The *OsMPK20-4* gene, which has a strong basal level expression in untreated healthy leaves, remained unchanged upon challenging with wounding, jasmonic acid (JA), salicylic acid (SA), ethylene (ET), NaCl and sucrose. The expression was slightly up-regulated by abscisic acid (ABA), H_2_O_2_, drought but more drastically by heavy metals and low temperature (12°C). However, *OsMPK20* expression was down-regulated at 37°C and by UV-C irradiation [28]. *OsMPK20* is also inducible by exogenous ABA treatment and *Magnaporthe grisea* infection and is associated with host cell death [19]. Most of the studies involving group D members of rice MAPKs have been limited to mRNA expression level and no interacting proteins have been identified.

In this study, we conducted a yeast two-hybrid analysis of OsMPK20-4 to find out its interacting partners in order to gain more insights into the rice MAPK pathway. Defying the linear nature of MAPK pathway, OsMPK3 (a member of group A MAPK) was found to be an interacting partner of OsMPK20-4 in yeast two-hybrid screens. Further *in vitro* and *in planta* experiments confirmed the specificity of the interaction. Interestingly, both the proteins were shown to enhance the plant immunity in tobacco against *Pseudomonas syringae* pv. *tabaci* infection. The two proteins were also observed to localize in the stomatal guard cells upon infection reflecting their possible role in stomatal defense. The current work showing the physical interaction between OsMPK20-4 and OsMPK3 opens a new dimension to the MAPK signaling research.

## Results

### OsMPK20-4 interacts with OsMPK3 in yeast

**Figure 1:**
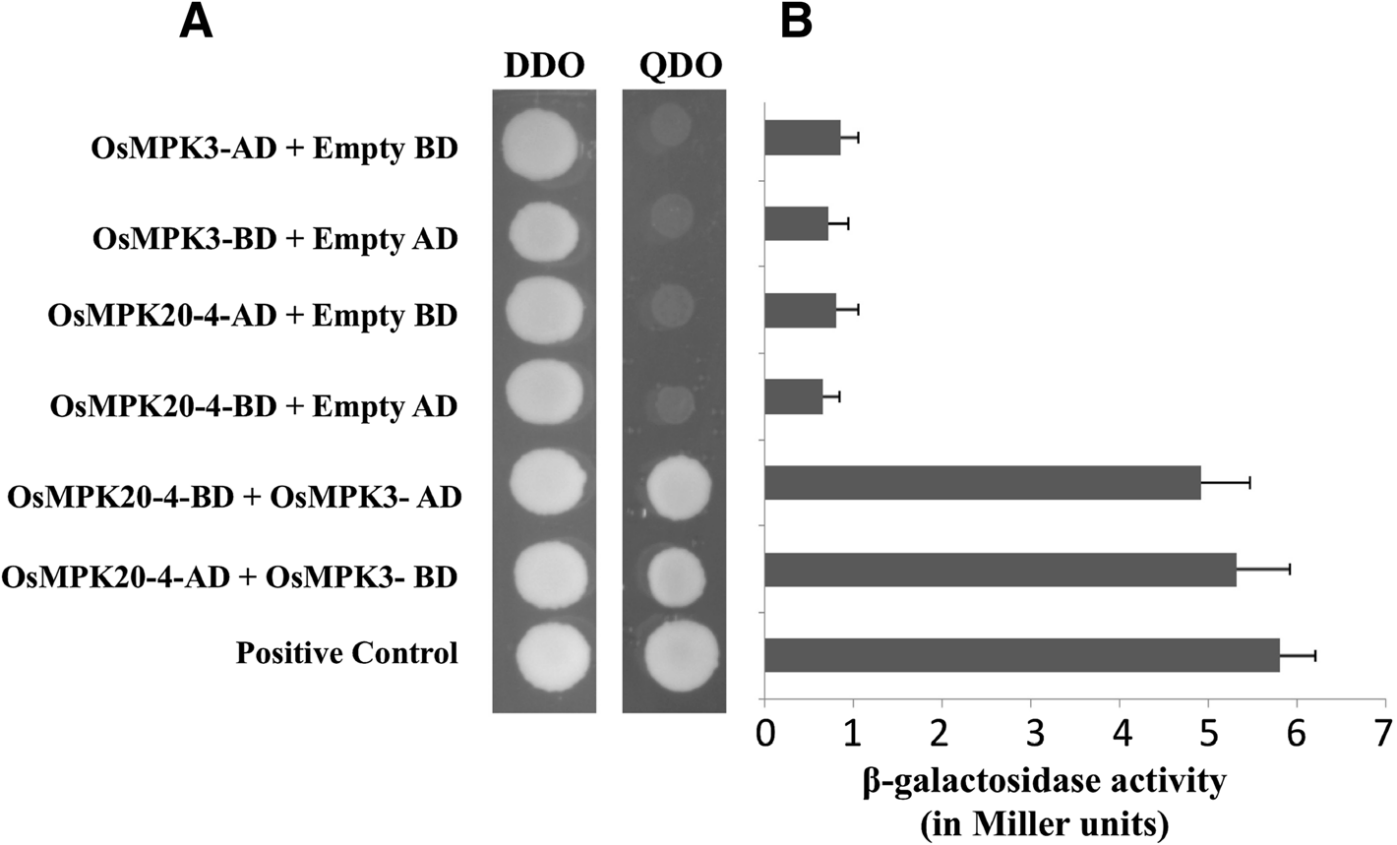
**Interaction of OsMPK20-4 with OsMPK3 by yeast two-hybrid screening. (A)** Reporter yeast strain AH109 was co-transformed with the recombinant pGBKT7 and pGADT7 encoding the listed gene constructs. Transformants were selected on SD [-leu-trp] double dropout (DDO) media and the interaction was checked on SD [-trp-leu-ade-his] quadruple dropout (QDO) media. **(B)** β-galactosidase assay showing the *lacZ* reporter gene expression by using ONPG as substrate. Liquid cultures of AH109 reporter strain transformed with the mentioned constructs were checked for β-galactosidase activity by adding ONPG and reading the absorbance at OD = 420 nm.AH109 co-transformed with SV40 large T antigen in pGADT7 vector and p53 in pGBKT7 vector served as positive control of protein interaction in all the experiments.

A yeast two-hybrid (Y2H) screen of cold treated (4°C) *Oryza sativa* cDNA library was initially performed to identify proteins that may interact with OsMPK20-4. After screening approximately 4 × 10^6^ transformants with OsMPK20-4 bait, eleven positive clones were identified. Sequence analysis of the clones revealed that one of them encoded an OsMAPK later identified to be OsMPK3. Subsequently, a full length cDNA clone of OsMPK3 was isolated and cloned in both yeast AD (activation domain) and BD (binding domain) vectors. The interaction was validated by performing one to one protein-protein interaction between full length clones of OsMPK20-4 and OsMPK3. The interaction was also observed after swapping the vectors between OsMPK20-4 and OsMPK3 (Figure 1A).

Since OsMPK3 and OsMPK20-4 interaction was positive on nutritional selection media (-ade, -his, -leu, -trp) as well as with Mel1 reporter gene expression, Lac Z reporter gene expression was confirmed by using ONPG as substrate. AH109 strain of yeast cotransformed with OsMPK3 and OsMPK20-4 yielded a high β- galactosidase activity whereas the other transformants with combination of OsMPK3 or OsMPK20-4 and empty AD or BD vectors have low or negligible amounts of β- galactosidase activity as compared to the positive interactions (Figure 1B).

### The interaction between OsMPK20-4 and OsMPK3 is specific

**Figure 2:**
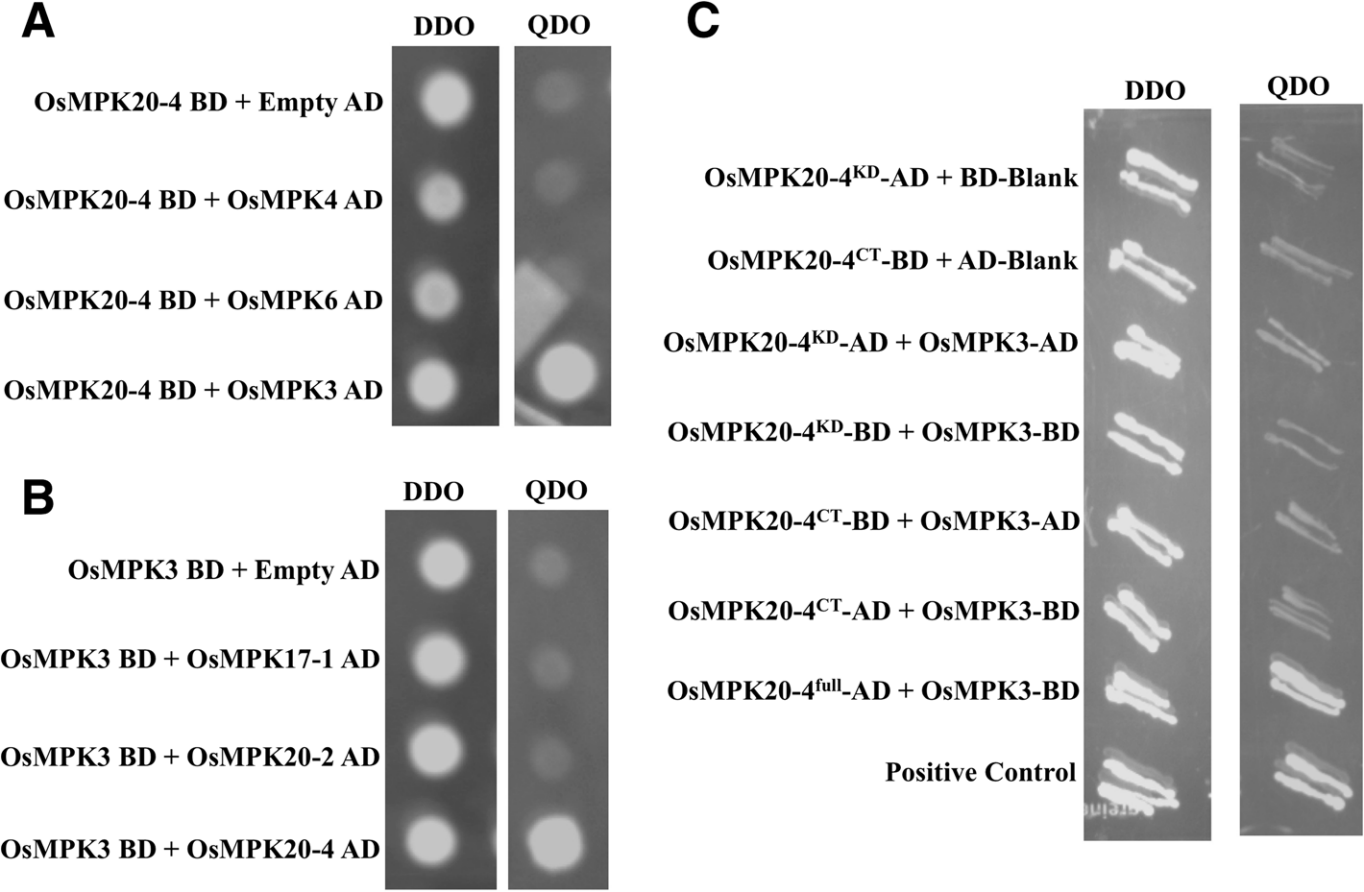
**Specificity of interaction between OsMPK20-4 and OsMPK3. (A)** Reporter yeast strain AH109 was co-transformed with the recombinant *OsMPK20-4* in pGBKT7 and close relatives of *OsMPK3* including *OsMPK4* and *OsMPK6* in pGADT7. **(B)** OsMPK3 in pGBKT7 was transformed into AH109 along with close relatives of OsMPK20-4 including OsMPK17-1 and OsMPK20-2. Transformants were selected on SD [-leu-trp] DDO media and the interaction was checked on SD [-trp-leu-ade-his] QDO media. **(C)** Full length OsMPK20-4 is required for efficient interaction with OsMPK3. AH109 yeast reporter strain was co-transformed with deletion constructs of OsMPK20-4, OsMPK20-4^KD^ containing first 358 amino acids and OsMPK20-4^CT^ containing the long C-terminal region of 211 amino acids in both AD and BD vectors. Transformants were selected on DDO and the interaction was checked on QDO. AH109 co-transformed with SV40 large T antigen in pGADT7 vector and p53 in pGBKT7 vector served as positive control in all the experiments.

In order to prove that the interaction between OsMPK20-4 and OsMPK3 is specific, a targeted one to one yeast two-hybrid assay was carried out for OsMPK20-4 and OsMPK3 against the phylogenetically close members of OsMPK3 and OsMPK20-4 respectively. The close relatives of OsMPK20-4 included OsMPK20-2 and OsMPK17-1, all group D MAPKs and that of OsMPK3 included OsMPK6 and OsMPK4 from group A and B MAPKs respectively [5]. The full length clones of OsMPK20-4, OsMPK20-2 and OsMPK17-1 in AD vectors were used for Y2H analysis against OsMPK3 in BD vector. Similarly the full length clones of OsMPK3, OsMPK4 and OsMPK6 in AD vectors were used against OsMPK20-4 in BD vector for Y2H assay. In both the cases only the combination of OsMPK20-4 and OsMPK3 either in AD or BD vector showed growth on nutritional selection media (-ade, -his, -leu, -trp) reflecting the specificity of the interaction (Figure 2, A-B).

The group D MAPKs lack C-terminal common docking (CD) domain that functions as a docking site for MAPKKs, instead they have a relatively long C-terminal region [19]. The next question asked was whether the relatively long C- terminal domain of OsMPK20-4 is responsible for the interaction with OsMPK3. For this, the C-terminal 211 amino acid long stretch of OsMPK20-4 (OsMPK20-4^CT^) was cloned in AD and BD vectors and used against OsMPK3 in a targeted Y2H assay. Also the portion of OsMPK20-4 containing the first 358 amino acids of kinase domains (OsMPK20-4^KD^) but lacking the C-terminal tail of 211 amino acids was also cloned in AD and BD vectors and used for interaction with OsMPK3 in Y2H study. In both the cases no interaction or a very weak interaction was observed suggesting the interaction is not solely mediated by long C-terminal amino acid stretch but requires a complete OsMPK20-4 for efficient interaction (Figure 2C).

### Confirmation of the interaction between OsMPK20-4 and OsMPK3 using *in planta* Co-immunoprecipitation and FRET assays

**Figure 3:**
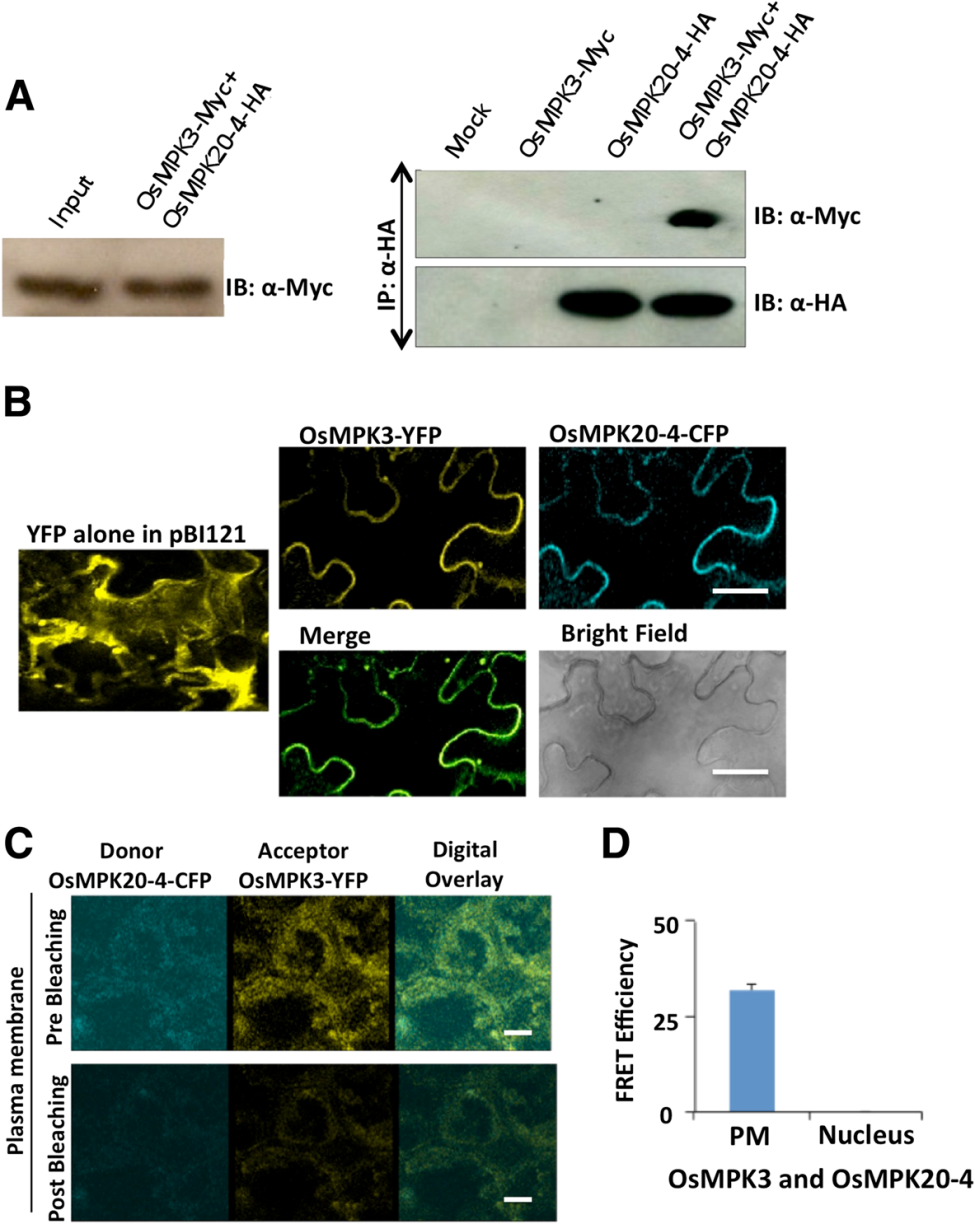
***In planta *interaction of OsMPK20-4 with OsMPK3. (A)** HA tagged OsMPK20-4 and Myc tagged OsMPK3 proteins transiently transformed individually or together in tobacco leaves were used for co-immunoprecipitation experiment. The protein from pCAMBIA1302 empty vector infiltrated leaves was used as negative control (Mock). First IP (immunoprecipitation) was carried out using anti-HA antibody then immunoblots (IB) of the precipitated proteins was carried out using anti-c-Myc and anti-HA antibodies. IBs were developed using HRP based chemiluminescent substrate. **(B)** Sub-cellular co-localization studies were conducted using YFP tagged OsMPK3 and CFP tagged OsMPK20-4 proteins. Localization of the indicated tagged proteins in transiently transformed tobacco leaves was observed using YFP and CFP filters of confocal laser scanning microscope (Leica AOBS system). Bars = 20 μm. **(C)** Fluorescence resonance energy transfer assay of the tagged proteins was conducted post 48 hr of transient transformation in tobacco leaves. Cyan and yellow are pseudo colors representing CYP and YFP respectively. Pictures were taken by confocal microscope (TCS SP5; Leica). Bars = 20 μm. **(D)** FRET efficiency (%) as observed in PM (plasma membrane) and nucleus (n = 3).

To further confirm the interaction between OsMPK20-4 and OsMPK3, co-immunoprecipitation (Co-IP) assay was performed. OsMPK3 with MYC tag and OsMPK20-4 with HA tag in pCAMBIA1302 binary vector were transiently transformed either independently or in combination (1:1) in *Nicotiana tabacum* leaves by agro-infiltration. After 48 hours, the *in planta* expression of the respective transcripts was checked (Additional file 1) and the proteins isolated were used for Co-IP assay. The expression of the proteins used as input control was checked by immunoblot (IB) with anti-Myc and anti-HA antibodies tagged to OsMPK3 and OsMPK20-4, respectively (Additional file 2). Immunoprecipitation of the isolated proteins was carried out using anti-HA antibody followed by immunoblot of the precipitated proteins using anti-c-Myc antibody. OsMPK3 was only detected in the anti-HA immunoprecipitates from proteins of co-transformed leaf tissues infiltrated with both OsMPK20-4-HA and OsMPK3-MYC (1:1) reflecting the interaction of the two proteins (Figure 3A).

The subcellular localization and subsequent co-localization of OsMPK20-4 and OsMPK3 was performed in tobacco leaves by transient transformation using agro-infiltration method. OsMPK20-4 tagged with CFP and OsMPK3 tagged with YFP cloned in pBI121 binary vector system were transiently transformed into *Nicotiana tabacum* leaves by agro-infiltration method. After confirming the presence of respective transcripts (Additional file 1), the leaves were observed under confocal laser scanning microscope under appropriate absorbance filters to check the localization of the fluorescent tagged proteins. The proteins were found to be co-localized on the peripheries of the cells and both the fluorescent signals were completely superimposable upon merging the images (Figure 3B). The fluorescence resonance energy transfer acceptor bleaching (FRET-AB) method was carried out to obtain the *in planta* evidence for the interaction of OsMPK3 and OsMPK20-4. The recombinant constructs of OsMPK3 carrying YFP tag and OsMPK20-4 carrying CFP tag were transiently transformed into tobacco leaves by agro- infiltration. Cells were further analysed for FRET-AB analysis. OsMPK3 was found to interact with OsMPK 20–4 *in planta* predominantly at the plasma membrane (Figure 3C and 3D).

### OsMPK20-4 and OsMPK3 are not phosphorylation targets of each other

**Figure 4:**
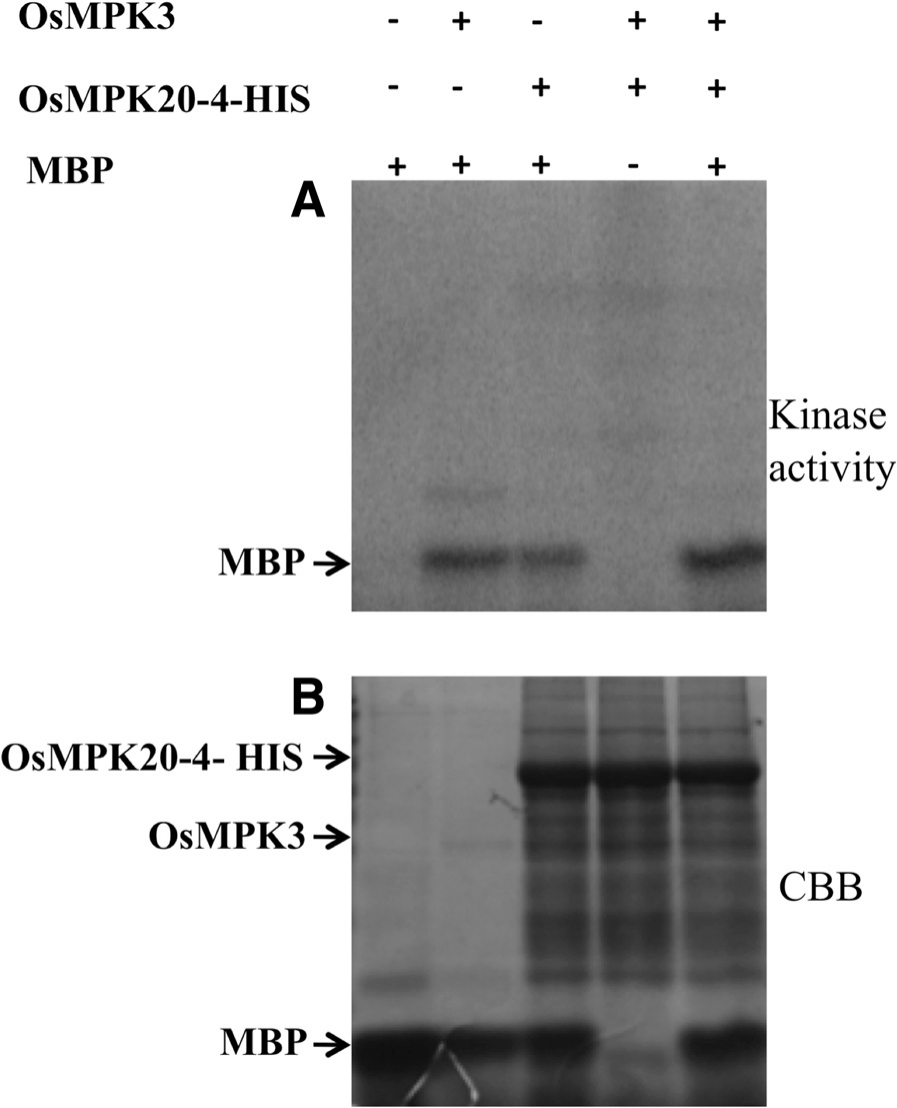
**OsMPK20-4 and OsMPK3 interaction is phosphorylation independent. ***In vitro* phosphorylation assay with bacterially expressed tag free OsMPK3 and His-tagged OsMPK20-4 showed the two proteins do not phosphorylate each other. The activities of the two kinases are shown by using the general MAPK substrate, MBP. The upper panel shows the autoradiograph while the lower panel shows the Coomassie stained protein of the same gel.

OsMPK20-4 and OsMPK3 have three and one putative [S/TP] sites respectively (Source: NetPhosK.1 web server) which are considered as the minimal consensus motif for MAPK phosphorylation [4]. Hence it was sought to further examine whether the interaction between two proteins is followed by phosphorylation event. To address the problem, bacterially expressed tag-free OsMPK3 (Additional file 3A) and His-tagged OsMPK20-4 (Additional file 3B) were used for an *in vitro* kinase assay in presence of kinase buffer containing radiolabelled [γ-^32^P] ATP. Even though both the proteins could independently phosphorylate myelin basic protein (MBP, an artificial substrate for MAPKs) they failed to phosphorylate each other reflecting the interaction may not be leading to phosphorylation of either of the protein (Figure 4). Interestingly, relatively strong phosphorylation of MBP was observed when both OsMPK3 and OsMPK20-4 were included together in the assay.

### OsMPK20-4 and OsMPK3 interaction show coordinated role in plant defense

**Figure 5:**
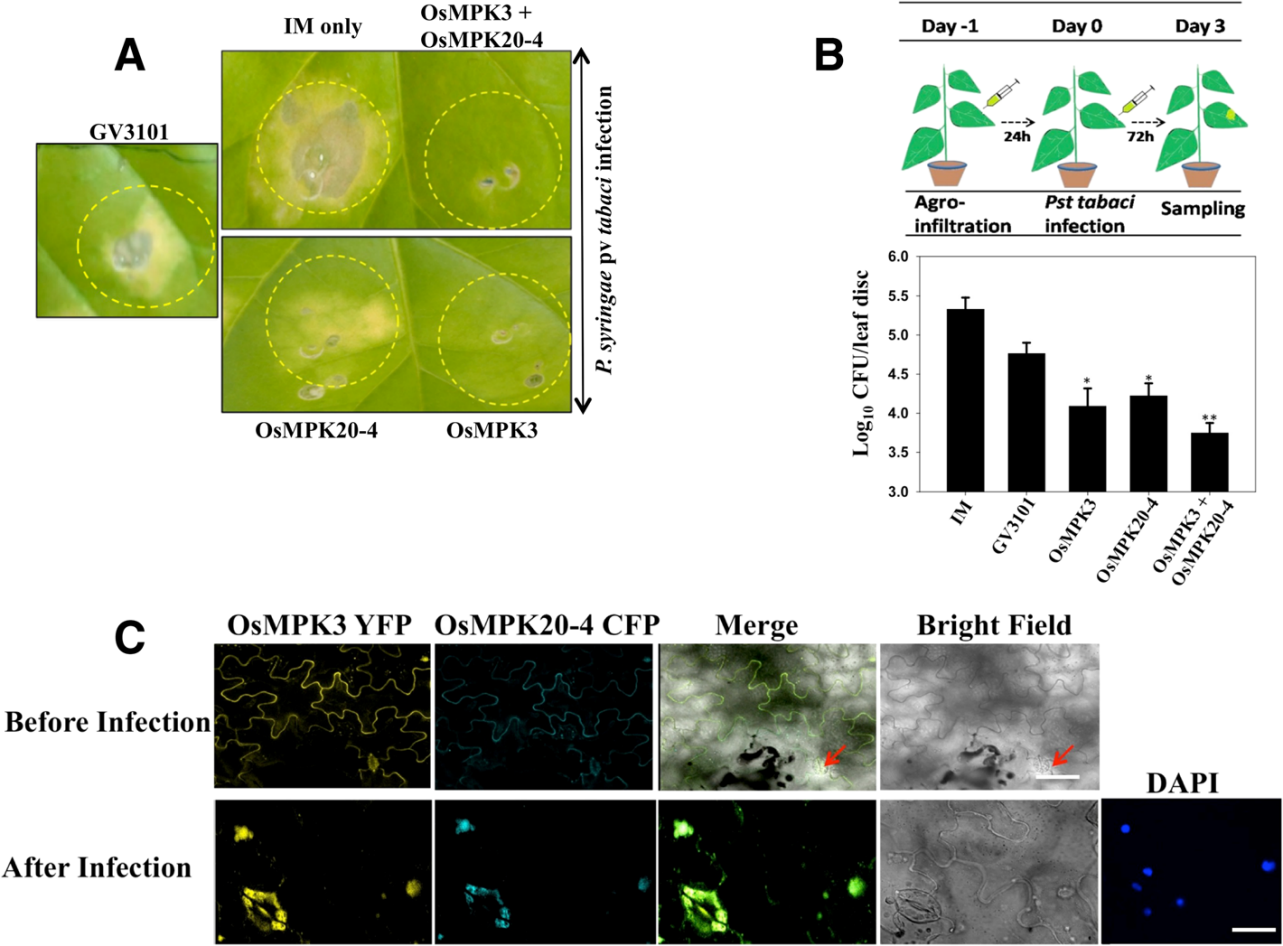
**OsMPK20-4 and OsMPK3 have role in plant defense. (A)** Both OsMPK20-4 and OsMPK3 give resistance against the disease. Tobacco leaves infiltrated with mock (IM only), *Agrobacterium* strain GV3101, OsMPK20-4-HA and OsMPK3-Myc in 1:1 ratio were challenged with *P. syringae* pv *tabaci* (OD_600_ 0.1) infection after 24 hours of agroinfiltration. Photographs were taken after 72 hours of infection. **(B)** OsMPK20-4 and OsMPK3 play synergistic role in plant defense. OsMPK20-4-HA and OsMPK3-Myc induced resistance was determined according to the schematic representation (upper panel), and the growth of *P. syringae* pv *tabaci* was plotted as log cfu/leaf disc (lower panel). Three independent experiments were conducted and the representative figure is shown. *t* test *P* values: * < 0.05, ** < 0.01. **(C)** OsMPK20-4 and OsMPK3 interaction plays role in stomatal defense. Upper panel shows the localization of the shown proteins before *P. syringae* pv *tabaci* infection. Lower panel shows the sudden translocation of both the rice MAPKs into the nuclei and stomatal guard cells after 24 h of infection. The pictures were taken from confocal laser scanning microscope (Leica AOBS system). Bars = 20 μm.

It has been previously established that rice OsMPK3 is involved in disease resistance response [20] and OsMPK20-4 is induced by *Magnaporthe grisea* infection and is associated with host cell death [19]. Also there existed a correlated gene expression of both OsMPK3 and OsMPK20-4 in rice under certain biotic challenges (Additional file 4) reflecting a concerted role of these proteins in plant defense. Hence, we sought to investigate whether the interaction of the two proteins affect infection in plants. For this, tobacco leaves were transiently transformed either with *OsMPK20-4/OsMPK3* individually or in combination and were challenged with *Pseudomonas syringae* pv. *tabaci.* After 72 hours, leaves expressing *OsMPK20-4*, *OsMPK3* or both showed an increased resistance to bacterial infection in comparison to mock treated leaves (Figure 5A). For controls, the leaves were independently infiltrated with *Agrobacterium* strain GV3101 carrying empty vector and infiltration medium (IM) only. Also the bacterial colony counting revealed that the leaves expressing both OsMPK20-4 and OsMPK3 show more disease resistance in comparison to individual proteins (Figure 5B). The observations indicate that the two interacting proteins act in a concerted manner in providing disease resistance response.

Fluorescent microscopy of CFP tagged OsMPK20-4 and YFP tagged OsMPK3 showed peripheral localization of the proteins. But interestingly through preliminary image analysis it was found that both OsMPK20-4 and OsMPK3 underwent transient translocation from the cell peripheries to the nuclei and stomatal guard cells following *Pseudomonas syringae* pv. *tabaci* infection (Figure 5C). This reflects that OsMPK20-4 and OsMPK3 besides being key players in general plant stress might have a role in stomatal defense.

## Discussion

In this study, we report the discovery of a novel interaction between two rice mitogen activated protein kinases OsMPK20-4 and OsMPK3. Although the interactions of MAPKs with their upstream MAPK kinases and downstream substrates are well known, there existed no previous report of two plant MAPKs physically interacting with each other. This finding adds one more, yet interesting dimension to the already complex plant MAPK signaling cascade. OsMPK20-4 is one of the elusive members of comparatively less studied but a very important rice MAPK signaling component. OsMPK20-4, a group D rice MAPK was initially characterized as OsWJUMK [28] in various abiotic stresses. It was observed to be induced by cold and heavy metal stress but not by wounding or jasmonic acid (hence the name Wound and Jasmonic acid Uninduced MAP Kinase). In another study high expression levels of OsMPK20-4 were found upon infection by rice blast fungus [19]. In a quest to find the interacting partners of OsMPK20-4, we carried out a yeast two-hybrid (Y2H) screening and identified OsMPK3 as the potential interacting partner (Figure 1A). The interaction was found to be specific as neither of them interact with each others phylogenetically close relatives (Figure 2A-B). It was also confirmed that the unusually long C-terminal portion of OsMPK20-4 (characteristic of group D MAPKs) involved in upstream kinase interaction, is not solely involved in the interaction with OsMPK3 (Figure 2C). The interaction of OsMPK20-4 and OsMPK3 was verified *in planta* by CoIP followed by FRET assay (Figure 3). Earlier two tobacco MAPKs, WIPK (*Arabidopsis* ortholog AtMPK3) and SIPK (*Arabidopsis* ortholog AtMPK6) were shown to functionally interact with each other during *Phytophthora* infection [29], but there was no report of direct physical interaction between two MAPKs. SIPK identified as the kinase involved in regulating WIPK gene expression, providing an indirect clue that MAPKs may interplay among themselves in regulating plant defense response.

The proteins were localized in the periphery of the cells which is in agreement with the observations made for *Arabidopsis* AtMPK3, an ortholog of OsMPK3 [30]. Singh et al. [31] in their report observed that OsMPK3 (named as OsMPK5) is localized both in nucleus and cytoplasm. The discrepancy in the localization of OsMPK3 in both the cases seems to be due to the difference in the biological systems and the experimental procedures used. The use of different expression systems in these studies might be another reason for the differences in protein localization. Also it is well known that MAP kinases follow a dynamic pattern depending on the physiological state of the cell and the nature of the protein they are interacting with [30,32,33]. However it can not be ruled out that co-localization and subsequent interaction of the two proteins may also occur due to overexpression owing to the fact that 35S promoter was used to drive the protein expression. The co-localization of the two proteins was deduced from merging of two individual fluorescent signals as documented for plant CDPKs [34]. Interestingly, positive FRET assay indicated *in planta* interaction of the two proteins and the FRET efficiency was observed in plasma membrane only. It may, therefore, be concluded that the two proteins interact on the cell peripheries. It is also known that OMTK1 (oxidative stress-activated MAP triple-kinase 1) a MAPKKK from alfalfa plays a MAPK scaffolding role by binding and selectively activating the alfalfa MAPK, MMK3 and functions in activation of H_2_O_2_-induced cell death in plants [35]. It can also be speculated that OsMPK20-4 acts as a scaffolding protein in binding and subsequently activating OsMPK3.

There existed a correlated gene expression of OsMPK20-4 and OsMPK3 under biotic stresses in rice MPSS database providing a hint of their concerted role in biotic stress (Additional file 4). Transient transformation of OsMPK20-4-HA and OsMPK3-Myc in tobacco leaves individually showed marked decrease in *P. syringae* pv. *tabaci* bacterial count validating the observations made by Song and Goodman [20] for OsMPK3 (Figure 5). The results contradict the earlier observations showing negative role of OsMPK3 (earlier named as OsMAPK5) in modulating PR gene expression and broad-spectrum disease resistance [22]. However, recently OsMPK3 was reported to positively regulate the JA signaling pathway and plant resistance to a chewing herbivore in rice [36] Also, a positive regulation of WIPK and SIPK genes against *Pseudomonas cichorii* – a bacterial pathogen was shown in *Nicotiana benthamiana*[37]. Resistance to the pathogen was compromised in the plants in which both the genes were silenced. As OsMPK3 is the ortholog of WIPK, transient expression of OsMPK3 might lead to the increase in resistance against pathogen and hence positive role in tobacco defense responses. OsMKK4–OsMPK3/OsMPK6 cascade is also shown to be involved in both positive and negative regulation of defense responses in rice [38]. Our data suggests that the two interacting proteins act in a coordinated manner in providing disease resistance response against *P. syringae*.

Plant MAPKs are known to exhibit a dynamic change in their localization while responding to biotic as well as abiotic stresses [33]. Nuclei and stomatal guard cells are two important sites for generating potential combat machinery for the infection [39]. Ozone treatment induced the translocation of *Arabidopsis* AtMPK3 and Pep-13 (*Phytophthora* spp. elicitor) treatment of parsley cells induced the translocation and accumulation of PcMPK6 into the nucleus [30,32]. *Arabidopsis* AtMPK3 and heteromeric G-protein, GPA1 are known to act in a parallel signaling pathways during ABA mediated stomatal closure [15]. In addition to having a role in stomatal guard cell movements, AtMPK3 is involved in active prevention of bacterial infection through stomata. In consent with the above observations, the translocation of both OsMPK20-4 and OsMPK3 into the nuclei and stomatal guard cells upon infection was observed. The concurrent movement of both of the proteins reflects a role of their interaction in generating stomatal defense response. The functions of OsMPK3 and AtMPK3 are conserved whereas OsMPK20-4 acts as a translocation enhancing protein.

## Conclusion

In the present study a novel interaction between two MAPKs, OsMPK20-4 and OsMPK3 has been reported. The interaction is phosphorylation independent and requires the full length proteins for efficient interaction. The interaction is required for the coordinated plant defense response against bacterial pathogen, *Pseudomonas syringae* and is also thought to play role in stomatal defense. Overall, the addition of this new link into the already complex MAPK pathway can further lead to understand the actual signaling mechanism behind various physiological responses.

## Methods

### Yeast two-hybrid screening

Total RNA was isolated from two week old rice plants exposed to cold stress (4°C) for 2 hours using Trizol reagent according to manufacturer’s instruction (Invitrogen, UK). First strand cDNA synthesis was performed using BD SMART III oligonucleotide and CDS III primer as per manufacturer’s instruction (Clontech). Second strand synthesis and amplification was performed using LD-PCR with 5′ and 3′ PCR primers. The amplified double stranded cDNA was purified with a BD CHROMA SPINTM TE-400 column. GAL4 AD fusion library was produced by cotransforming yeast AH109 strain with BD SMART dscDNA and SmaI-linearized pGADT7-Rec vector. The bait was produced by in-frame cloning of *OsMPK20-4* in pGBKT7 vector (Clontech) to form OsMPK20-4 GAL4 BD fusion protein. Now the yeast reporter strain AH109 was cotransformed with the dscDNA, pGADT7-Rec and pGBKT7-*OsMPK20-4* plasmids by PEG/LiAc method according to the manufacturer’s instruction (Clontech). After 24 h of growth in SD -trp- leu (DDO, double dropout medium) at 30°C, the colonies were subsequently plated onto SD-trp-leu-ade-his medium (quadruple drop-out (QDO) medium). Positive interactions were selected on the basis of expression of HIS3, ADE2, and MEL1 reporter genes by growth on QDO medium. All the positive colonies were picked for screening analysis. Positive clones were confirmed by DNA sequencing.

Full length genes *OsMPK20-4* and *OsMPK3*, in-frame cloned in pGADT7 & pGBKT7 vectors (Clontech) and transformed in AH109 yeast strain were selected on QDO medium at 30°C for one to one interaction.

β- galactosidase assay was performed by monitoring the *LacZ* reporter gene expression directly on nutritional selection plates by addition of ONPG to the liquid culture which was rapidly freeze/ thawed as per manufacturer’s instructions (Clontech). As β- galactosidase accumulates in the medium, it hydrolyses ONPG to O-nitrophenol which is spectrophotometrically determined at 420 nm.

Full length clones of *OsMPK17-1*, *OsMPK20-2*, *OsMPK4* and *OsMPK6* were cloned in pGADT7 vector (Clontech) and cotransformed along with either *OsMPK3* or *OsMPK20-4* in pGBKT7 vector (Clontech) into yeast AH109 and selected on QDO at 30°C.

The plasmids pGBKT7:*OsMPK20-4*^*KD*^ and pGBKT7:*OsMPK20-4*^*CT*^ were generated by subcloning the N-terminal kinase domains of OsMPK20-4 (amino acid 1–358) and C-terminal region (amino acid 359–569) into the EcoRI/BamHI sites of the yeast pGBKT7 and pGADT7 vectors (Clontech) such that inserts were cloned in-frame with the DNA-binding domain and activation domain of the yeast GAL4 transcriptional regulator respectively. The list of all the primers used in the study is given in Additional file 5.

### Co-immunoprecipitation assay

For co-immunoprecipitation, OsMPK20-4 and OsMPK3 were tagged with N-terminal HA and MYC tags by in-frame cloning in pGADT7 and pGBKT7 vectors (Clontech) respectively. The OsMPK20-4-HA and OsMPK3-MYC tag were amplified by PCR from pGADT7 and pGBKT7 vectors and two additional tags were introduced using primers OsMPK3-MYC-pcmF and OsMPK3-MYC-pcmR for MYC tag and OsMPK20-4-HA-pcmF and OsMPK20-4-HA-pcmR for HA tag (Additional file 5). The cassette was transferred to pCAMBIA 1302 binary vector system. The binary vectors were transformed into *Agrobacterium* strain GV3101. The fusion proteins were coexpressed in *Nicotiana tabacum* leaves using *Agrobacterium* transient infiltration method [35]. After 48 hours proteins were isolated by using 50 mM HEPES-KOH (pH 7.5), 5 mM EDTA, 5 mM EGTA, 1 mM DTT, 10 mM Na_3_VO_4_, 10 mM NaF, 50 mM β-glycerolphosphate, 1 mM PMSF, 10% (v/v) glycerol, 0.1% Nonidet P-40, 2.5% PVPP and protease inhibitor cocktail (Sigma). Immunoprecipitation (IP) of the extracted proteins was carried out using anti-HA antibody and then immunoblot of the precipitated proteins was carried out using anti-c-Myc antibody. Briefly 200 μg protein was incubated with 2 μg anti-HA antibody (Santa Cruz Biotechnology) and incubated at 4°C for 4–6 hours. Then 40 mg ProteinA Sepharose™ 6 MB beads (GE Healthcare) were added and incubated overnight at 4°C with constant rocking. Beads after washing were directly separated on 10% SDS-PAGE and then transferred to Hybond™-C extra membrane (Amersham). The membrane was incubated with anti-c-Myc primary antibody (1:5000 dilution) (Santa Cruz Biotechnology) and then with anti-mouse goat secondary antibody (1:10000 dilution). The membrane was developed using Immobilon™ Western Chemiluminescent HRP substrate (Millipore).

### Co-localization assays and fluorescence resonance energy transfer analysis

*OsMPK20-4* and *OsMPK3* were in-frame cloned in pECFP-N1 and pEYFP-N1 vectors (Clontech) to produce C-terminal CFP and YFP fluorescent fusion proteins (primer sequences mentioned in Additional file 5). The cassette was also cloned into pBI121 binary vector (Clontech). The binary vectors were transformed into *Agrobacterium* strain GV3101. The fusion proteins were coexpressed in *Nicotiana tabacum* leaves by Agrobacterium transient infiltration method [40] using hypodermic needleless syringe. After 72 hours the leaf discs and after 24 hours protoplasts were observed under Confocal scanning microscope (Leica TCS SP2 AOBS system) using CFP and YFP filters. The fluorescent excitations using Argon laser source at 514 nm for YFP and 435 nm for CFP while emissions of 527 nm and 475 nm were used. For FRET analysis the leaves were observed after 24 and 48 hours post transient transformation. The FRET experiments were conducted as per manufacturer’s instructions and FRET efficiency was recorded in more than three cells at one time [41]. Cyan fluorescent protein (CFP) was excited by argon laser at a wavelength of 458 nm and emission was between 465 nm and 505 nm, whereas YFP was excited at a wavelength of 512 nm and emission was between 525 nm and 600 nm. The fluorescence detected from CFP or YFP proteins were recorded. The confocal microscope (TCS SP5; Leica) was used for FRET experiments.

### *In vitro* phosphorylation assay

*OsMPK20-4* was in-frame cloned in pET21c(+) expression vector (Novagen) to get C-terminal His-tag and was transformed into competent *E. coli* BL21 cells. The protein was induced by 1 mM IPTG and solubilized into supernatant fraction by using *IBS*™ buffer kit (G Biosciences, USA). The protein was purified by QIAexpressionist™ protein purification system (Qiagen) using Ni-NTA agarose beads. On the other hand, *OsMPK3* was cloned into pPAL7 expression vector (Biorad) for tag free protein production. Protein was induced by 1 mM IPTG and purified using Profinity eXact™ protein purification system (Biorad). *In vitro* kinase assay was performed as described [42] with slight modifications. Briefly, 5 μg of sample was mixed with reaction buffer to give a final volume of 15 μl containing 25 mM Tris-Cl (pH 7.5), 10 mM MgCl_2_, 5 mM MnCl_2_, 1 mM DTT, 1 mM β-glycerolphosphate, 1 μM Na_3_VO_4_, 0.5 mg/ml MBP, 25 μM ATP and 1 μCi [γ-^32^P]ATP. Incubation at 30°C was stopped after 30 minutes by addition of 10 μl of 2× SDS sample buffer. Samples were boiled at 95°C for 5 minutes and then separated on 12% SDS-PAGE gel. Kinase activities were visualized using phosphor imager (Typhoon, Phosphor Storage System).

### Tobacco infiltration and infection studies

*Nicotiana tabacum* plants were grown at 25°C, 14 hour light cycle in green house. Four- to six- week old plants were used for *Agrobacterium* mediated transient transformation. Briefly, *Agrobacterium* strain GV3101 carrying different constructs was grown overnight at 28°C in YEB medium (yeast extract 1 g/l, beef extract 5 g/l, peptone 5 g/l, sucrose 5 g/l and MgSO_4_ 0.491 g/l; final pH 7.0 with NaOH) with appropriate antibiotics. Cells were collected by centrifugation (4000 g), resuspended to OD_600_ of 1.0 in infiltration medium (10 mM MES pH 5.7, 10 mM MgCl_2_ and 150 μM acetosyringone) and infiltrated into fully expanded leaves using needleless syringe. After 24 hours *Pseudomonas syringae* pv *tabaci* infection was given at areas overlapping the initial *Agrobacterium* infiltration. Bacterial inoculum was prepared by overnight growth at 28°C in LB medium with 50 mg/L tetracyclin, centrifuged and adjusted to OD_600_ of 0.1 in 10 mM MgCl_2_.

Bacterial colony counting assay was performed after 72 hours of infection. To assess bacterial population, three 10-mm^2^ leaf discs were harvested from inoculated areas and ground in 1 ml of 10 mM MgCl_2_, diluted and plated to determine the log_10_CFU/leaf disc as described [43] with slight modifications. Briefly, after syringe infiltration, three 10 mm^2^ leaf discs were ground in a tube and 100 μl of three fold serial dilution was spotted onto LB plates with 50 mg/L tetracyclin. Colonies were counted 48 h after plating.

## Abbreviations

ABA: Abscisic acid; AD: Activation domain; BD: Binding domain; CD Domain: Common docking domain; CFP: Cyan fluorescent protein; Co-IP: Coimmunoprecipitation; DDO: Double drop out; FRET: Fluorescence resonance energy transfer; JA: Jasmonic acid; MAPK: Mitogen activated protein kinase; MBP: Myelin basic protein; ONPG: Ortho-nitrophenyl-β-galactoside; OsWJUMK: *Oryza sativa* wound and jasmonic-acid uninduced MAP kinase; QDO: Quadruple drop out; SA: Salicylic acid; Y2H: Yeast two-hybrid; YFP: Yellow fluorescent protein.

## Competing interests

The authors declare that they have no competing interests.

## Authors’ contributions

AHS performed all experiments and wrote the first draft of the manuscript. AHS and AKS analyzed the data. SKJ expressed tag-free OsMPK3 protein and helped AHS in pathogen infection analysis. RR and DPW cloned various MAPK genes in AD and BD yeast vectors. PS performed FRET assay. RR, SKJ, DPW and PS participated in writing of the manuscript. AKS conceived the idea, supervised the project and wrote the final draft of the manuscript. All authors read and approved the final manuscript.

## Supplementary Material

Additional file 1 **Expression of transiently transformed gene constructs in *Nicotiana tabacum *leaves.** To confirm the expression of agro-infiltrated gene constructs in tobacco leaves, semi-quantitative RT PCR of the cDNA preparations from infiltrated regions was performed using OsMPK3 and OsMPK20-4 specific primers. Click here for file

Additional file 2 **Protein expression of transiently transformed OsMPK3-Myc and OsMPK20-4-HA tagged genes in *Nicotian tabaccum. ***Immunoblot (IB) was performed using anti-c-Myc antibody and anti-HA antibodies 48 h post transformation. The same extracts were used as input for the data shown in Figure 3A. Click here for file

Additional file 3 **Bacterial expression and purification of OsMPK3 and OsMPK20-4. *A***, OsMPK3 was cloned in pPAL7 (BioRad), transformed and induced by 1 mM IPTG in BL21 cells and finally purified tag free ***B***, OsMPK20-4 was cloned into pET21c expression vector (Novagen), transformed into BL21 cells, induced by 1 mM IPTG and purified using Ni NTA agarose beads (Qiagen). Click here for file

Additional file 4 **Rice MPSS database showing correlative expression of OsMPK20-4 and OsMPK3 under biotic stress.** In 60 day mature rice leaves, roots and meristematic tissues, a correlated gene expression of the two OsMAPKs was observed under *M. greisea* infection. 9LA, 9LC, 9LD symbolize infection on mature leaves. 9ME symbolizes meristematic tissues while 9RO and 9RR symbolize mature roots. Similarly FLA and FLB symbolize F1-hybrid mature leaves while FRO and FRR show infection response of F1-hybrid mature roots. Source: http://mpss.udel.edu/in9311/mpss_index.php. Click here for file

Additional file 5 List of primer sequences used to clone the studied genes. Click here for file
