# Supplementary figures and images for: Interaction between two rice mitogen activated protein kinases and its possible role in plant defense
Source: BMC Plant Biol. 2013 Aug 28;13:121. doi: 10.1186/1471-2229-13-121 (PMC3765826; doi:10.1186/1471-2229-13-121)

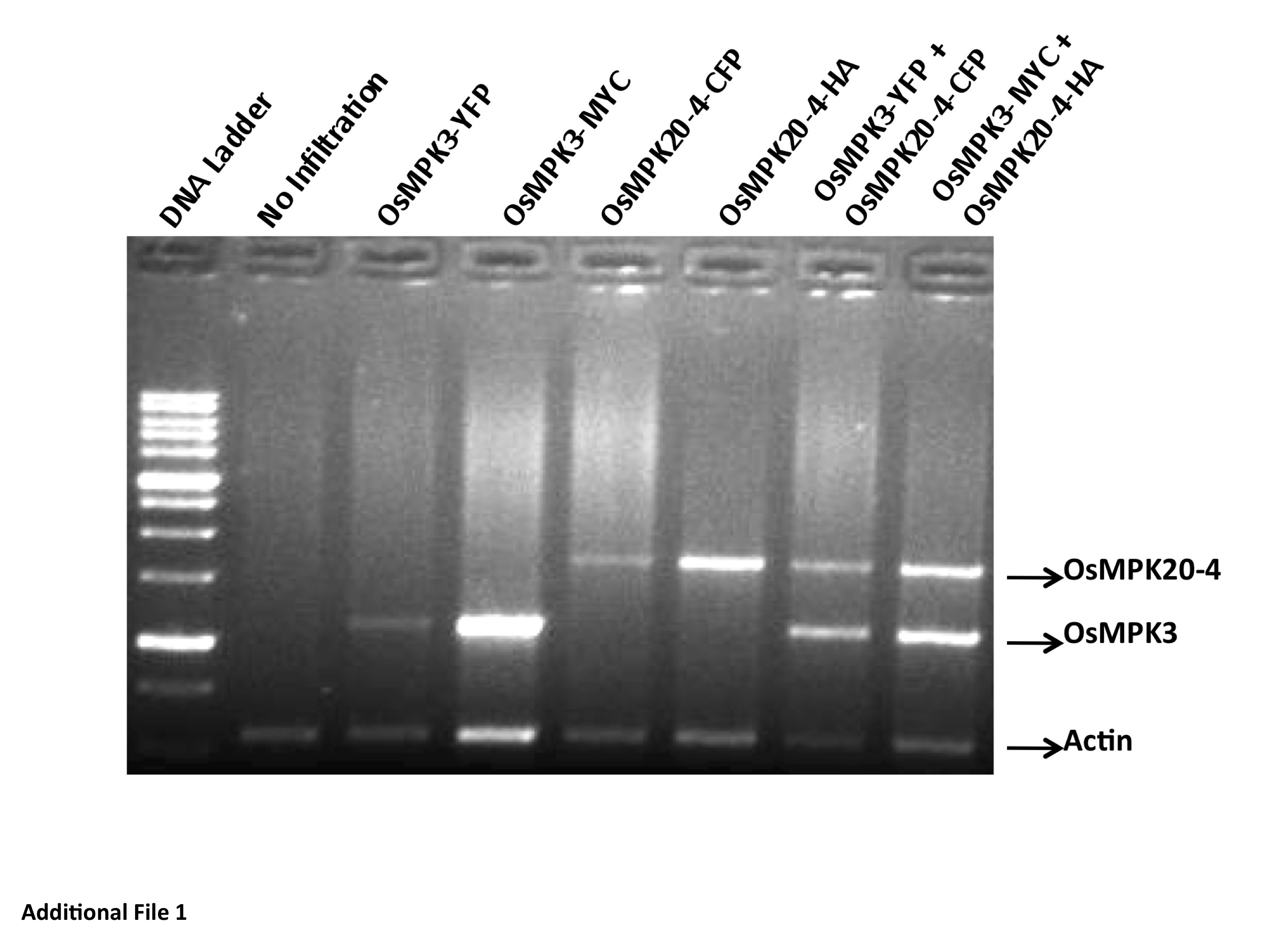

Supplement: Additional file 1 — Expression of transiently transformed gene constructs in Nicotiana tabacum leaves. To confirm the expression of agro-infiltrated gene constructs in tobacco leaves, semi-quantitative RT PCR of the cDNA preparations from infiltrated regions was performed using OsMPK3 and OsMPK20-4 specific primers. [file 1471-2229-13-121-S1.jpeg]

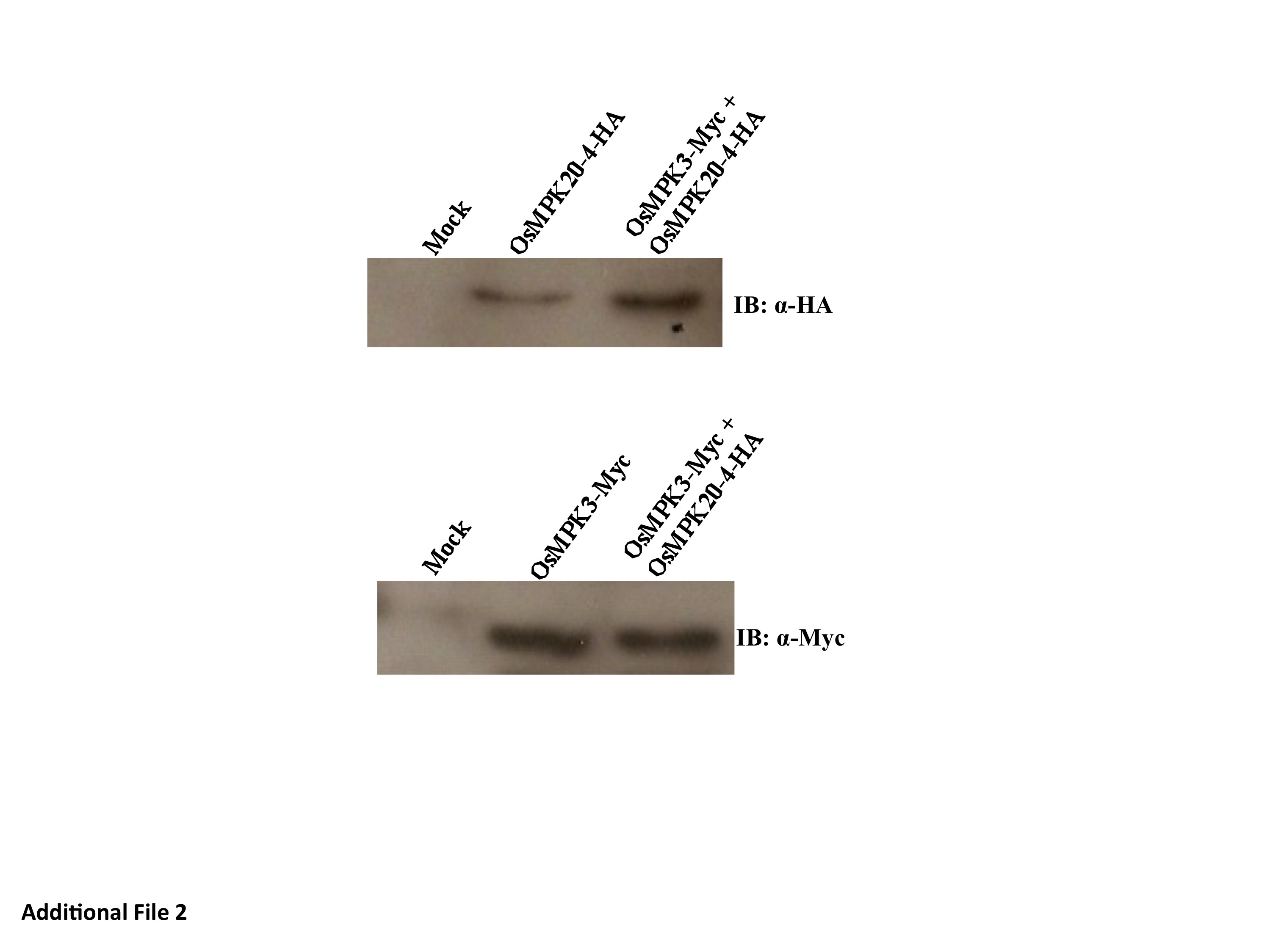

Supplement: Additional file 2 — Protein expression of transiently transformed OsMPK3-Myc and OsMPK20-4-HA tagged genes in Nicotian tabaccum. Immunoblot (IB) was performed using anti-c-Myc antibody and anti-HA antibodies 48 h post transformation. The same extracts were used as input for the data shown in Figure 3A. [file 1471-2229-13-121-S2.jpeg]

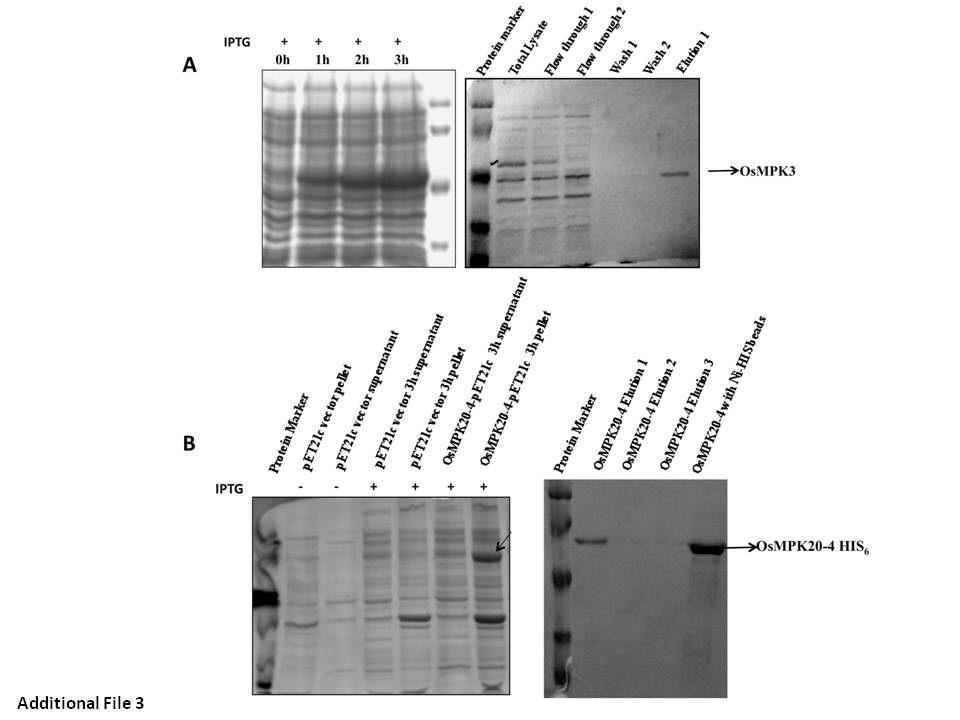

Supplement: Additional file 3 — Bacterial expression and purification of OsMPK3 and OsMPK20-4. A, OsMPK3 was cloned in pPAL7 (BioRad), transformed and induced by 1 mM IPTG in BL21 cells and finally purified tag free B, OsMPK20-4 was cloned into pET21c expression vector (Novagen), transformed into BL21 cells, induced by 1 mM IPTG and purified using Ni NTA agarose beads (Qiagen). [file 1471-2229-13-121-S3.jpeg]

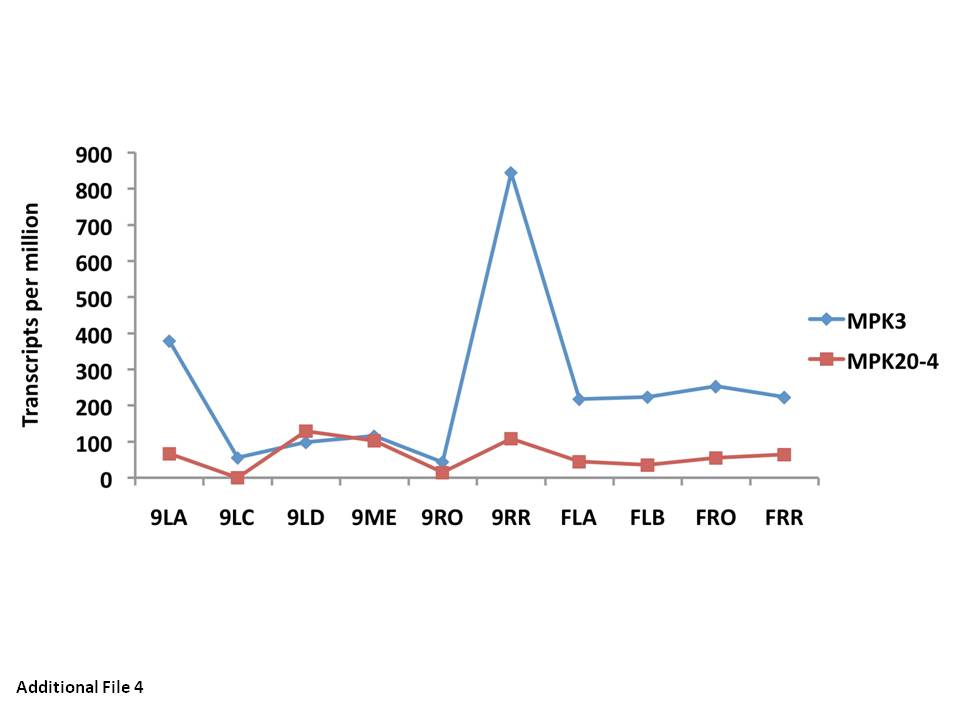

Supplement: Additional file 4 — Rice MPSS database showing correlative expression of OsMPK20-4 and OsMPK3 under biotic stress. In 60 day mature rice leaves, roots and meristematic tissues, a correlated gene expression of the two OsMAPKs was observed under M. greisea infection. 9LA, 9LC, 9LD symbolize infection on mature leaves. 9ME symbolizes meristematic tissues while 9RO and 9RR symbolize mature roots. Similarly FLA and FLB symbolize F1-hybrid mature leaves while FRO and FRR show infection response of F1-hybrid mature roots. Source: http://mpss.udel.edu/in9311/mpss_index.php. [file 1471-2229-13-121-S4.jpeg]

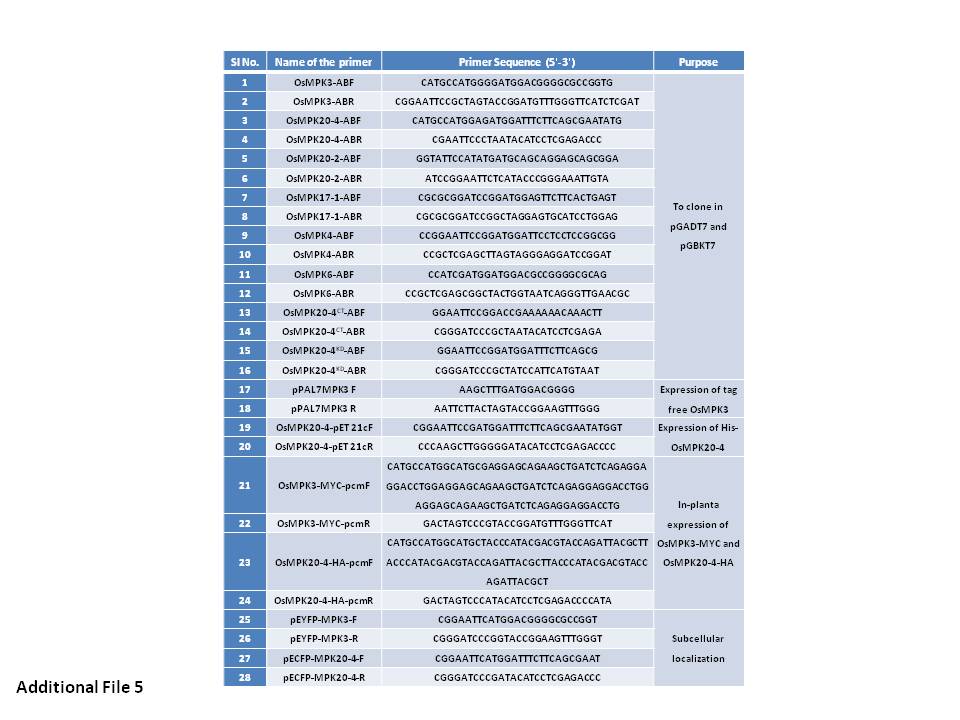

Supplement: Additional file 5 — List of primer sequences used to clone the studied genes. [file 1471-2229-13-121-S5.jpeg]
